# Quantitative proteomic analysis of single or fractionated radiation-induced proteins in human breast cancer MDA-MB-231 cells

**DOI:** 10.1186/2045-3701-5-2

**Published:** 2015-02-03

**Authors:** Mi-Hyoung Kim, Seung-Youn Jung, Jiyeon Ahn, Sang-Gu Hwang, Hee-Jong Woo, Sungkwan An, Seon Young Nam, Dae-Seog Lim, Jie-Young Song

**Affiliations:** Division of Radiation Cancer Research, Korea Institute of Radiological & Medical Sciences, 215-4 Gongneung-dong, Nowon-gu, Seoul 139-706 Korea; Laboratory of Immunology, College of Veterinary Medicine, Seoul National University, Seoul, Korea; Department of Microbiological Engineering, Kon-Kuk University, Seoul, Korea; Radiation Health Institute, Korea Hydro & Nuclear Power Co., Ltd, Seoul, Korea; Department of Applied Bioscience, CHA University, Gyeonggi-do, Korea

**Keywords:** Breast cancer, Proteomics, Radiation therapy, SILAC

## Abstract

**Background:**

Radiotherapy is widely used to treat cancer alone or in combination with surgery, chemotherapy, and immunotherapy. However, damage to normal tissues and radioresistance of tumor cells are major obstacles to successful radiotherapy. Furthermore, the immune network around tumors appears to be connected to tumor progression and recurrence.

**Methods:**

We investigated the cytosolic proteins produced by irradiated tumor cells by using a quantitative proteomic approach based on stable isotope labeling by amino acids in cell culture. MDA-MB-231 breast cancer cells were treated with a single or fractionated 10 Gray dose of ^137^Cs γ-radiation, which was selected based on cell viability.

**Results:**

Radiation-induced proteins were differentially expressed based on the fractionated times of radiation and were involved in multiple biological functions, including energy metabolism and cytoskeleton organization. We identified 46 proteins increased by at least 1.3-fold, and high ranks were determined for cathepsin D, gelsolin, arginino-succinate synthase 1, peroxiredoxin 5, and C-type mannose receptor 2.

**Conclusion:**

These results suggest that a number of tumor-derived factors upregulated by γ-radiation are promising targets for modulation of the immune response during radiation treatment.

**Electronic supplementary material:**

The online version of this article (doi:10.1186/2045-3701-5-2) contains supplementary material, which is available to authorized users.

## Background

Radiation therapy (RT) is one of the most common treatments for cancer and can be provided alone or, frequently, in combination with surgery, chemotherapy, or immunotherapy [1, 2]. Depending on the type of cancer, 50–90% of cancer patients undergo RT during the course of their illness, with the highest percentages in cases of non-small-cell lung cancer, prostate, breast, and colon cancer [3, 4]. However, RT is associated with acute or chronic side effects such as injury to normal tissues, fatigue, nausea/vomiting, diarrhea, and intestinal bleeding. The effect of radiation on tumor cells varies greatly according to the type of radiation, total dose, dose rate, and time of testing post-exposure [4–7]. Therefore, the reasons for RT failure are multiple and vary, and major causes include inadequate vascular supply (hypoxia), cancer stem cells, and novel mutations [8]. Furthermore, tumors can survive in complex systems, including heterogeneous tumor cells, tumor-associated cells, normal cells, and immune cells, rather than as a simple mass of malignant cells. Notably, tumor-associated immune cells produce various signals that are highly predictive of the efficiency of RT as well as cancer progression and recurrence [9].

In the last several years, stimulation of the immune system has been proposed to occur through immunogenic cell death (ICD) mediated mainly by danger signals from endogenous damage-associated molecular patterns (DAMPs) such as stress, damage or injury to tissues, and radiation. However, the putative beneficial and detrimental roles of DAMPs in cancer therapy are highly controversial [7, 9]. Profiling the factors derived by irradiated cancer cells could therefore provide new insights into the prediction of radiation responsiveness.

Breast cancer is the most prevalent malignancy in women and the second leading cause of cancer-related deaths in developed countries. RT is widely used as part of a tri-modal treatment with chemotherapy and surgery; however, approximately 50% of breast cancer patients have experienced malignant microfoci scattered throughout the breast tissue that can easily progress to metastatic breast cancer [10]. Therefore, new approaches are urgently needed, and multimodal combinatorial therapy is currently being investigated. To this end, we performed a massive quantitative proteomic analysis of irradiated human breast cancer cells (MDA-MB-231) based on stable isotope labeling by amino acids in cell culture (SILAC), which has emerged as one of the most powerful tools for accurate and robust quantitative proteomics, and liquid chromatography–mass spectrometry (LC-MS) [11–13].

In this study, we demonstrated that the tumor-derived proteins commonly increased by both single and fractionated radiation are involved in multiple biological functions. These results indicate promising ICD-associated candidates for cancer treatment that may predict and modify the response to RT.

## Methods

### Cell culture

The human breast cancer cell line MDA-MB-231 was purchased from American Type Culture Collection (ATCC, Manassas, VA, USA). The cells were maintained at 37°C in RPMI 1640 with 10% fetal bovine serum (FBS; Gibco BRL, Grand Island, NY, USA), 100 units/mL penicillin, and 100 μg/mL streptomycin in a humidified atmosphere with 5% CO_2_.

### Cell viability tests

Cell viability was assessed by trypan blue and MTT (3-[4,5-dimethylthiazol-2-yl]-2,5-diphenyltetrazolium bromide) assays (Sigma-Aldrich, St. Louis, MO, USA) according to the manufacturer’s recommendations. MDA-MB-231 cells were plated in 96-well plates (1 × 10^4^ cells/well) in triplicate for indicated times after irradiation. MTT (0.5 mg/mL) was added to each well for 3 h, and absorbance was measured at 540 nm using a microplate reader (Multiskan EX, Thermo LabSystems, Waltham, MA, USA). Additionally, to ascertain cell death, cells were incubated with 2.5 mg/mL propidium iodide (PI) for 5 min at room temperature and analyzed with a FACSCanto II flow cytometer (Becton Dickson, Franklin Lakes, NJ, USA) and Flowing Software (version 2.5.1; http://www.flowingsoftware.com/).

### Stable isotope labeling by amino acids in cell culture

MDA-MB-231 cells were maintained in l-lysine-depleted RPMI 1640 (Invitrogen, Grand Island, NY, USA) supplemented with 10% dialyzed FBS (Invitrogen, Carlsbad, CA, USA) and 0.1 mg/mL heavy [U-^13^C_6_] or light l-lysine (Invitrogen). Every 3–4 days, cells were split and media replaced with the corresponding light or heavy labeling medium. After approximately six doubling times, cells achieved almost 100% incorporation of amino acid isotopes. Cells grown with light l-lysine (1 × 10^6^ cells/100 mm dish) were exposed to 10 Gray (Gy) ^137^Cs γ-radiation (Gammacell 3000 Elan, MDS Nordion, Canada) and harvested after 48 h.

### Subcellular fractionation

An equal ratio (1:1) of treated and untreated MDA-MB-231 cells were mixed and fractionated using the ProteoJET™ Cytoplasmic and Nuclear Protein Extraction Kit K0311 (Fermentas, Canada) according to the manufacturer’s instructions. The efficacy of fractionation was determined via western blotting using GAPDH and Lamin A/C as the cytosolic and nuclear control proteins, respectively. BIOCON (Suwon, Korea) was conducted to identify proteins altered by irradiation.

### Database searching

The tandem mass spectra were extracted, and Sorcerer 3.4 beta2 (Sorcerer software 3.10.4, Sorcerer Web interface 2.2.0.r334) was used to deconvolute and de-isotope the charge states. All of the MS/MS samples were analyzed using Sequest (Thermo Fisher Scientific, San Jose, CA, USA; version 1.3.0.339) set up to search the IPI HUMAN 3.87 database (unknown version, 91464 entries) assuming trypsin digestion. Sequest was searched with a fragment ion mass tolerance of 0.8 or 1.0 Da and a parent ion tolerance of 10.0 PPM. The cysteine iodoacetamide derivative was specified in Sequest as a fixed modification. Deamidated asparagine and glutamine, ^13^C-lysine, ^13^C- and ^15^N-arginine, oxidized methionine, acetylated lysine, and phosphorylated serine, threonine, and tyrosine were specified as variable modifications.

### Quantitative data analysis

Scaffold Q+ (version 3.6.2, Proteome Software Inc., Portland, OR, USA) was used to validate MS/MS-based peptide and protein identifications. Peptide identifications were accepted if they were established at greater than 95.0% probability by the Peptide Prophet algorithm [14]. Protein probabilities were assigned by the Protein Prophet algorithm [15], and identifications were accepted if they were established at greater than 99.0% probability and contained at least two unique identified peptides. Experimentally acquired intensities were globally normalized across all acquisition runs. Individual quantitative samples were normalized within each acquisition run. Intensities for identified peptides were normalized within the assigned protein. The reference channels were normalized to produce a 1:1 fold change. All normalization calculations were performed using medians to multiplicatively normalize data.

### Western blot

Cells were lysed with RIPA buffer (50 mM Tris–Cl, pH 7.4, 1% NP-40, 150 mM NaCl, 1 mM EDTA) supplemented with protease inhibitors (1 mM phenylmethylsulfonyl fluoride, 1 μg/mL aprotinin, 1 μg/mL leupeptin, and 1 mM Na_3_VO_4_). Proteins from whole cell lysates were separated on 8–15% SDS-polyacrylamide gels and transferred to nitrocellulose membranes (Bio-Rad, Hercules, CA, USA). The membrane was blocked with 5% skim milk in Tris-buffered saline containing 0.1% Tween-20 (TBS-T) for 1 h and probed with primary antibodies overnight at 4°C. Primary antibodies for cathepsin D (CTSD), gelsolin (GSN), argininosuccinate synthase 1 (ASS1), and C-type mannose receptor 2 (MRC2) were obtained from Santa Cruz Biotechnology (Santa Cruz, CA, USA), and the primary antibody for GAPDH was obtained from AbFrontier (Seoul, Korea). After multiple washes, membranes were incubated with peroxidase-conjugated secondary antibodies, and immunoreactive bands were detected using enhanced chemiluminescence reagents according to the manufacturer’s recommendations (GE Healthcare, Little Chalfont, UK). Experiments were repeated at least three times.

## Results

### Radiation- induced dose-dependent cytotoxicity in MDA-MB-231 cells

**Figure 1:**
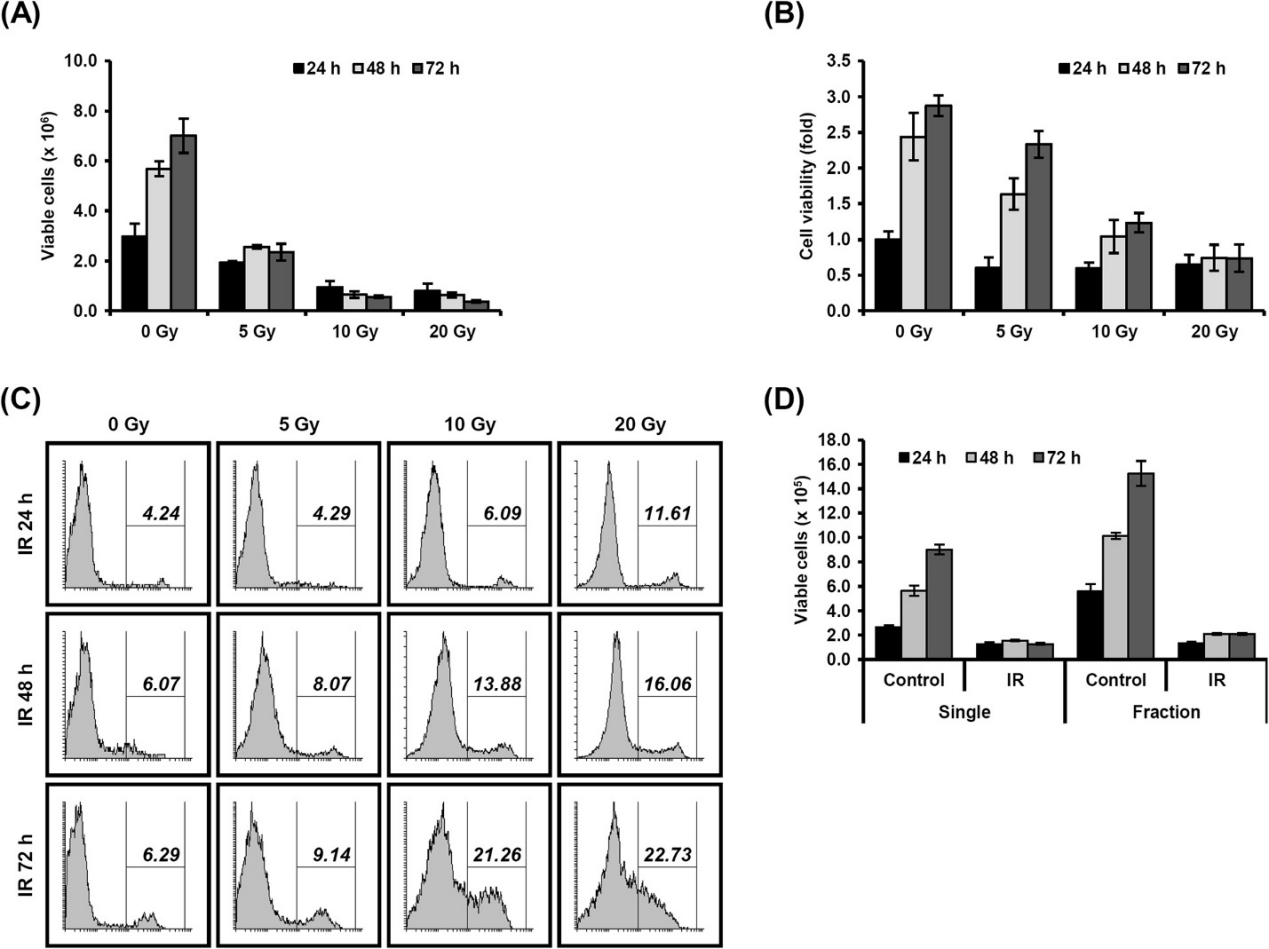
**Evaluation of cytotoxicity by radiation.** MDA-MB-231 cells were treated with the indicated dose of radiation and incubated for 24, 48, or 72 h. Cell viability was determined by trypan blue exclusion assay **(A)**, MTT assay **(B)**, and FACS analysis using propidium iodide (PI) fluorescent dye **(C)**. **(D)** MDA-MB-231 cells were treated with a single dose of radiation (10 Gy) or fractionated dose of radiation (2 Gy per day for 5 days). Viable cells were determined by trypan blue exclusion assay 24, 48, or 72 h after final irradiation.

Whereas apoptosis does not activate the immune response, necrotic cell death contributes to inflammation and pathophysiological function through release or secretion of diverse molecules [16]. Therefore, to identify the immune-related molecules produced by tumor cells in response to RT, we first investigated the appropriate radiation dose and harvesting time of MDA-MB-231 cells, highly aggressive and triple-negative breast cancer cells, by trypan blue exclusion (Figure 1A), MTT (Figure 1B), and PI staining (Figure 1C) assays. Exposure to ionizing radiation significantly decreased cell viability in a dose- and time-dependent manner. Cells exposed to radiation above 10 Gy continued to proliferate after 72 h in the MTT assay but not in the trypan blue exclusion assay, indicating that the methods possess different sensitivities to radiation-induced cell death. In accordance with the trypan blue assay, the percent cell death determined by PI staining increased after irradiation. Cells were harvested 48 h after 10 Gy of radiation, chosen to evoke radiation-mediated protein alterations but prevent extensive death, for further experiments. Along with the radiation dose, the effects of fractionated irradiated protocols on normal tissue damage and tumor-host interactions are also important in cancer treatment. Cell survival following single or fractionated irradiation seems to be similar in *in vitro* studies when the total doses are equal [17]. We also measured cell cytotoxicity after fractionated irradiation up to 10 Gy in 5 daily fractions by trypan blue exclusion (Figure 1D) for comparison with single-dose irradiation. We observed no differences in cell cytotoxicity between single- and fractionated-dose irradiation samples (Additional file 1: Figure S1).

### Quantitative proteomic analysis of radiation-induced proteins in MDA-MB-231 cells

**Figure 2:**
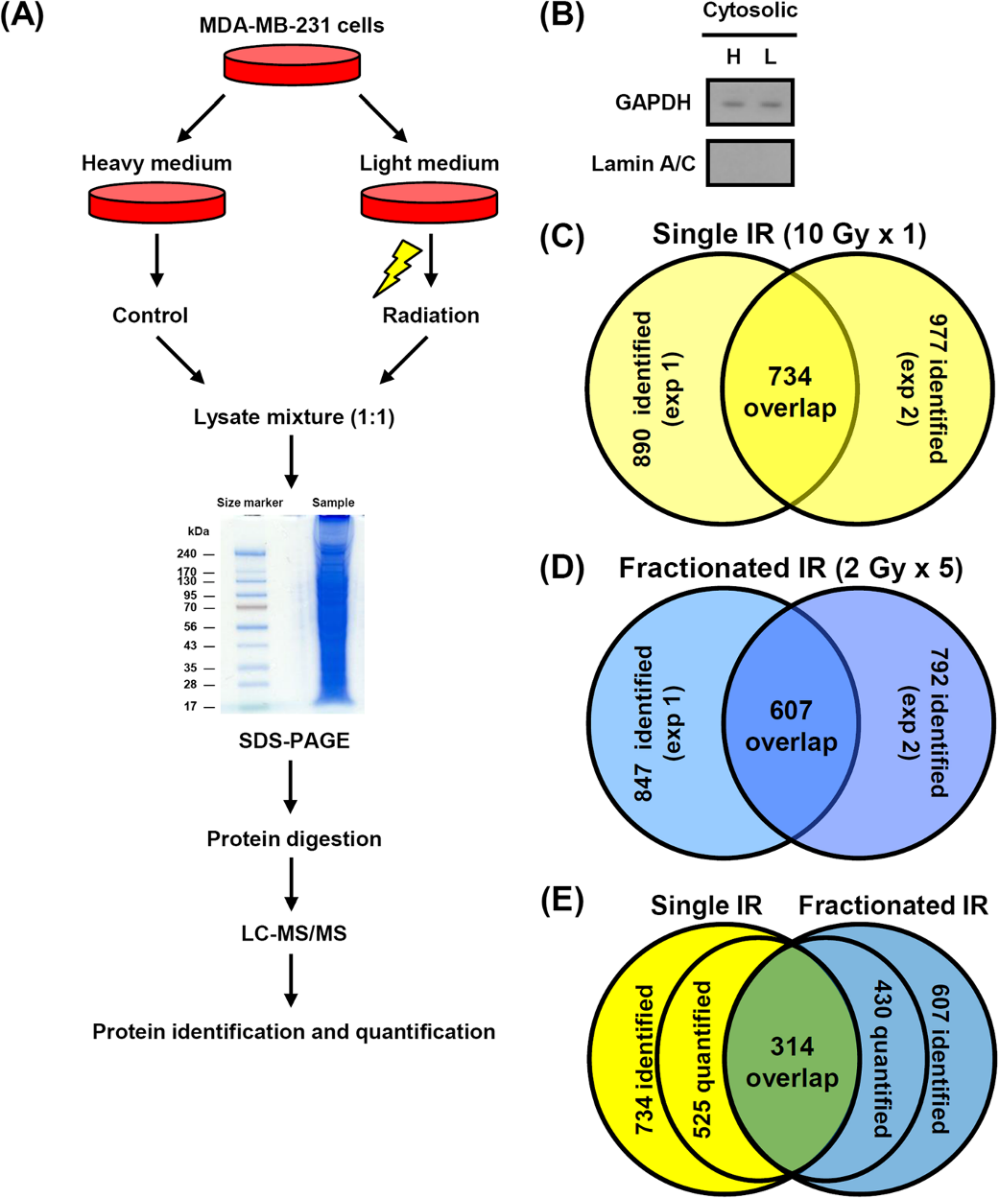
**Venn diagram summary of identified proteins by SILAC-based quantitative proteomics. (A)** Schematic workflow for profiling radiation-induced proteins via proteomic-based analysis. **(B)** The cytoplasmic lysates from SILAC-labeled cells were analyzed by western blotting to exclude contamination of nuclear extracts using GAPDH and Lamin A/C as cytosolic and nuclear control proteins, respectively. **(C)** 734 proteins were identified in the set of single dose experiments, and **(D)** 607 proteins were identified in the set of fractionated dose experiments. **(E)** Comparison of identified proteins from single or fractionated dose of irradiation.

Recently, several studies showed that cells exposed to fractionated radiation exhibit different signatures compared to those treated with a single dose of radiation [18–20]. Therefore, to quantitatively analyze proteome alterations in tumor cells treated with different fractionation regimes of radiation, SILAC-based proteomic analysis was performed. A schematic diagram of the experimental workflow is provided in Figure 2A. MDA-MB-231 cells in light media were treated with 10 Gy in a single dose (10 Gy × 1) or in multiple fractionated doses (2 Gy × 5) of radiation. Forty-eight hours after exposure, cytosolic proteins were isolated for analysis (Figure 2B). In duplicated experiments with single dose radiation, 890 and 977 proteins were identified, respectively, with 734 identified in both experiments and 525 quantified. In addition, 847 and 792 proteins were identified after multiple fractionated doses of radiation with 607 identified in both trials and 430 quantified. In total, 314 proteins from MDA-MB-231 cells were simultaneously quantified in both dosing strategies (Figure 2C–E).

### Classification of radiation-induced upregulated proteins

A change of 1.3–2.0-fold is generally used as a cut-off value for significance in SILAC proteomic approaches [13, 21]. Among the 314 proteins quantified, 46 increased at least 1.3-fold after radiation treatment (Additional file 2: Table S1). For clear comparison of the results, we classified the proteins upregulated at least 1.5-fold as common to both single and fractionated irradiation, single irradiation-specific-, or fractionated irradiation- specific (Table 1). Ionizing radiation induced the greatest increase in fibronectin 1 (FN1), CTSD, GSN, ASS1, and peroxiredoxin 5 (PRDX5) expression, but the increase in FN1 expression was not statistically significant (*P* = 0.1451).

**Table 1:** **Up-regulated proteins were identified by a SILAC-based proteomic approach**

no	Accession No	Gene symbol	Identified proteins	Fold	***P*** value ^*^	S 1	S 2	F 1	F 2
***Commonly up-regulated proteins***									
1	IPI00022418	FN1	Isoform 1 of Fibronectin	6.525	0.0000	1.7	1.9	17.7	4.8
2	**IPI00011229**	**CTSD**	**Cathepsin D**	**3.200**	**0.0000**	**2.9**	**3.2**	**3.4**	**3.3**
3	**IPI00026314**	**GSN**	**Isoform 1 of Gelsolin**	**2.375**	**0.0000**	**3**	**2.6**	**2**	**1.9**
4	**IPI00020632**	**ASS1**	**Argininosuccinate synthase**	**2.175**	**0.0007**	**2.3**	**2.1**	**2.4**	**1.9**
5	IPI00024915	PRDX5	Isoform Mitochondrial of Peroxiredoxin-5, mitochondrial	2.025	0.0606	2.9	1.8	1.5	1.9
6	IPI00019157	CSPG4	Chondroitin sulfate proteoglycan 4	1.900	0.3173	1.4	1	2	3.2
7	IPI00218474	ENO3	Isoform 1 of Beta-enolase	1.900	0.0145	1	1	4.6	1
8	**IPI00005707**	**MRC2**	**C-type mannose receptor 2**	**1.850**	**0.0074**	**1.3**	**1.2**	**2.5**	**2.4**
9	IPI00940829	NEDD4	Isoform 4 of E3 ubiquitin-protein ligase NEDD4	1.850	0.0004	1.3	1.3	2.4	2.4
10	IPI00021048	MYOF	Isoform 1 of Myoferlin	1.800	0.0000	1.8	1.7	2.4	1.3
11	IPI00021812	AHNAK	Neuroblast differentiation-associated protein AHNAK	1.800	0.0047	1.8	2.2	1.6	1.6
12	IPI00020557	LRP1	Prolow-density lipoprotein receptor-related protein 1	1.800	0.0000	1.4	1.4	1.8	2.6
13	IPI00021766	RTN4	Isoform 1 of Reticulon-4	1.725	0.0019	1.6	1.5	1.7	2.1
14	IPI00006663	ALDH2	Aldehyde dehydrogenase, mitochondrial	1.625	0.0262	2.1	1.8	1.4	1.2
***Single (10 Gy)***									
1	IPI00011229	CTSD	Cathepsin D	3.05		2.9	3.2		
2	IPI00026314	GSN	Isoform 1 of Gelsolin	2.8		3	2.6		
3	IPI00024915	PRDX5	Isoform Mitochondrial of Peroxiredoxin-5, mitochondrial	2.35		2.9	1.8		
4	IPI00020632	ASS1	Argininosuccinate synthase	2.2		2.3	2.1		
5	IPI00021812	AHNAK	Neuroblast differentiation-associated protein AHNAK	2		1.8	2.2		
6	IPI00006663	ALDH2	Aldehyde dehydrogenase, mitochondrial	1.95		2.1	1.8		
7	IPI00022418	FN1	Isoform 1 of Fibronectin	1.8		1.7	1.9		
8	IPI00021048	MYOF	Isoform 1 of Myoferlin	1.75		1.8	1.7		
9	IPI00021766	RTN4	Isoform 1 of Reticulon-4	1.55		1.6	1.5		
10	IPI00216694	PLS3	Plastin-3	1.55		1.6	1.5		
11	IPI00382844	ACO2	Aconitase (Fragment)	1.55		1.5	1.6		
12	IPI00013860	HIBADH	3-hydroxyisobutyrate dehydrogenase, mitochondrial	1.55		1.5	1.6		
13	IPI00018350	MCM5	DNA replication licensing factor MCM5	1.55		1.6	1.5		
14	IPI00098902	OGDH	2-oxoglutarate dehydrogenase, mitochondrial	1.5		1.5	1.5		
15	IPI00784156	AP2B1	Isoform 1 of AP-2 complex subunit beta	1.5		1.5	1.5		
16	IPI00456969	DYNC1H1	Cytoplasmic dynein 1 heavy chain 1	1.5		1.5	1.5		
***Fraction (2 Gy × 5)***									
1	IPI00022418	FN1	Isoform 1 of Fibronectin	11.25				17.7	4.8
2	IPI00011229	CTSD	Cathepsin D	3.35				3.4	3.3
3	IPI00218474	ENO3	Isoform 1 of Beta-enolase	2.8				4.6	1
4	IPI00019157	CSPG4	Chondroitin sulfate proteoglycan 4	2.6				2	3.2
5	IPI00005707	MRC2	C-type mannose receptor 2	2.45				2.5	2.4
6	IPI00940829	NEDD4	Isoform 4 of E3 ubiquitin-protein ligase NEDD4	2.4				2.4	2.4
7	IPI00020557	LRP1	Prolow-density lipoprotein receptor-related protein 1	2.2				1.8	2.6
8	IPI00216457	HIST2H2AA3	Histone H2A type 2-A	2.2				2.3	2.1
9	IPI00020632	ASS1	Argininosuccinate synthase	2.15				2.4	1.9
10	IPI00026314	GSN	Isoform 1 of Gelsolin	1.95				2	1.9
11	IPI00018398	PSMC3	26S protease regulatory subunit 6A	1.95				2.8	1.1
12	IPI00021766	RTN4	Isoform 1 of Reticulon-4	1.9				1.7	2.1
13	IPI00021048	MYOF	Isoform 1 of Myoferlin	1.85				2.4	1.3
14	IPI00293464	DDB1	DNA damage-binding protein 1	1.75				1.4	2.1
15	IPI00815770	SNX3	Isoform 1 of Sorting nexin-3	1.75				2.3	1.2
16	IPI00024915	PRDX5	Isoform Mitochondrial of Peroxiredoxin-5, mitochondrial	1.7				1.5	1.9
17	IPI00007682	ATP6V1A	V-type proton ATPase catalytic subunit A	1.7				1.7	1.7
18	IPI00021812	AHNAK	Neuroblast differentiation-associated protein AHNAK	1.6				1.6	1.6
19	IPI00010418	MYO1C	Isoform 2 of Myosin-Ic	1.6				1.8	1.4
20	IPI00000005	NRAS	GTPase NRas	1.6				1.6	1.6
21	IPI00006482	ATP1A1	Isoform Long of Sodium/potassium-transporting ATPase subunit alpha-1	1.6				1.4	1.8
22	IPI00023006	ACTC1	Actin, alpha cardiac muscle 1	1.6				2.1	1.1
23	IPI00221035	BTF3	Isoform 1 of Transcription factor BTF3	1.6				1.6	1.6
24	IPI00007402	IPO7	Importin-7	1.55				2.2	0.9
25	IPI00246058	PDCD6IP	Programmed cell death 6-interacting protein	1.55				1.7	1.4
26	IPI00026689	CDK1	Putative uncharacterized protein DKFZp686L20222	1.5				1.5	1.5
27	IPI00001960	CLIC4	Chloride intracellular channel protein 4	1.5				1.4	1.6

Proteins increased by an average of 1.5-fold or more are listed.

*Statistical comparisons were performed using the paired *z-*test, and experimentally verified proteins are represented in bold. S: single-dose irradiation, F; fractionated-dose irradiation.

**Figure 3:**
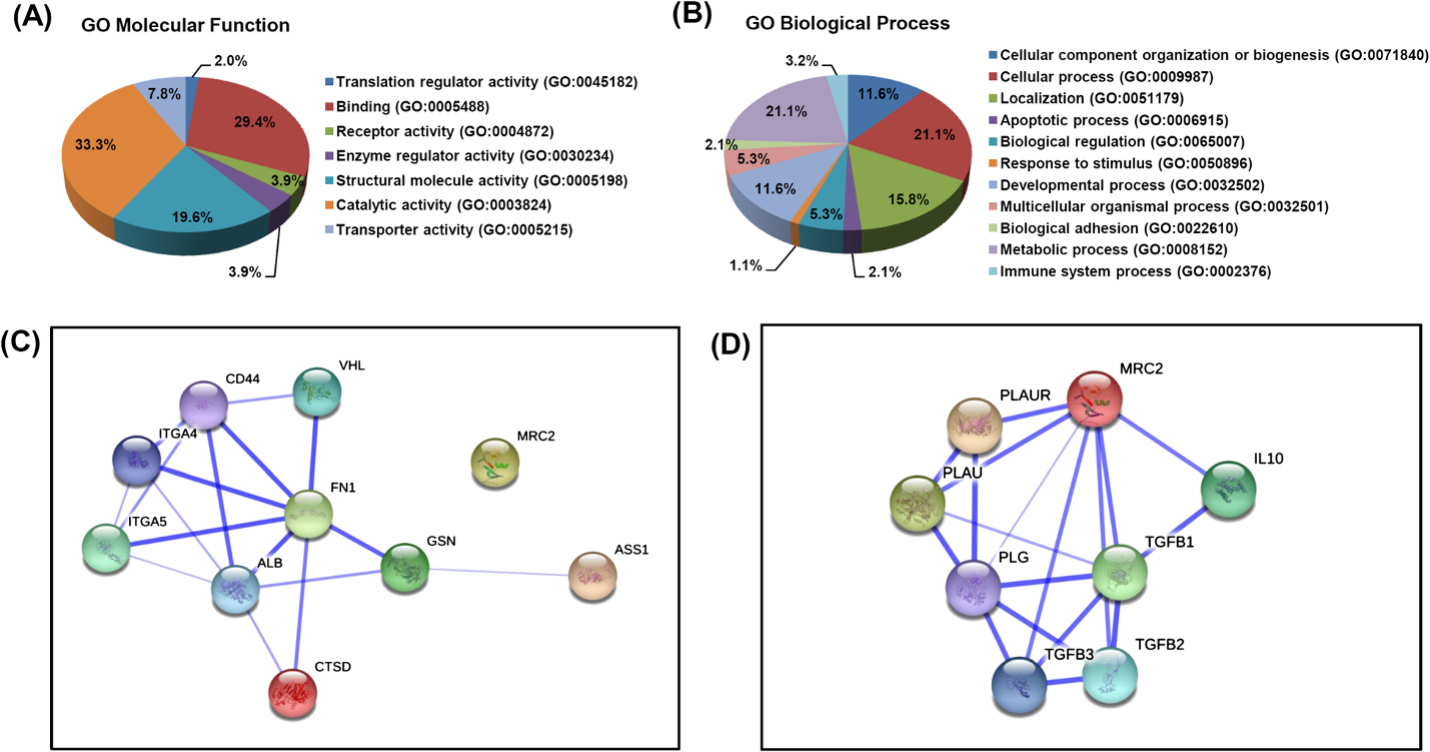
**Functional analysis for proteins upregulated after irradiation.** Gene ontology (GO) classification was performed according to molecular functions **(A)** and biological processes **(B)** using PANTHER (http://www.pantherdb.org/). Interaction network analysis of interesting proteins including FN1, CTSD, GSN, ASS1 **(C)**, and MRC2 **(D)** was performed using STRING (http://string-db.org).

Although most identified proteins were similarly increased in both single- and fractionated-dose radiation, some proteins such as FN1; chondroitin sulfate proteoglycan 4 (CSPG4); MRC2; neural precursor cell expressed, developmentally down-regulated 4 (NEDD4); and low-density lipoprotein receptor-related protein 1 (LRP1) were particularly increased by fractionated radiation. In contrast, single-dose radiation increased the expression of proteins, including GSN, AHNAK nucleoprotein, and aldehyde dehydrogenase 2 family, mitochondrial (ALDH2) to a greater extent than fractionated-dose radiation. Additionally, 53 proteins were decreased at least 1.3-fold by radiation, most of which were identified as ribosomal proteins (Additional file 2: Table S2). Gene ontology (GO) analysis was performed on upregulated proteins using Panther (version 9.0; http://www.pantherdb.org) according to the GO terms for molecular function and biological process [22] (Figure 3A and B). The upregulated proteins were significantly enriched in molecular functions of catalytic activity (33.3%), binding activity (29.4%), and structural and molecular activity (19.6%). With regard to biological processes, upregulated proteins were involved in metabolic processes (21.1%), cellular processes (21.1%), localization (15.8%), cellular component organization or biogenesis (11.6%), and developmental processes (11.6%).

### Interaction analysis of selected proteins

Among the top-ranked proteins in SILAC results, five interesting proteins were selected (FN1, CTSD, GSN, ASS1, and MRC2) based on *P* values and immunological activity potentials. STRING (version 9.1, http://string-db.org) was used to investigate the interaction potential of these proteins [13]. Interaction analysis identified close association of four proteins (FN1, CTSD, GSN, and ASS1; Figure 3C) and indicated potential interactions with cluster of differentiation 44 (CD44), integrin alpha 4 (ITGA4), and integrin alpha 5 (ITGA5), which are involved in cell-cell adhesion and immune response. Because MRC2 was not closely linked with the other four, the interaction network of MRC2 was separately verified (Figure 3D), identifying interactions with the plasminogen activator system and anti-inflammatory proteins such as transforming growth factor-beta (TGF-β) and interleukin-10 (IL-10).

**Figure 4:**
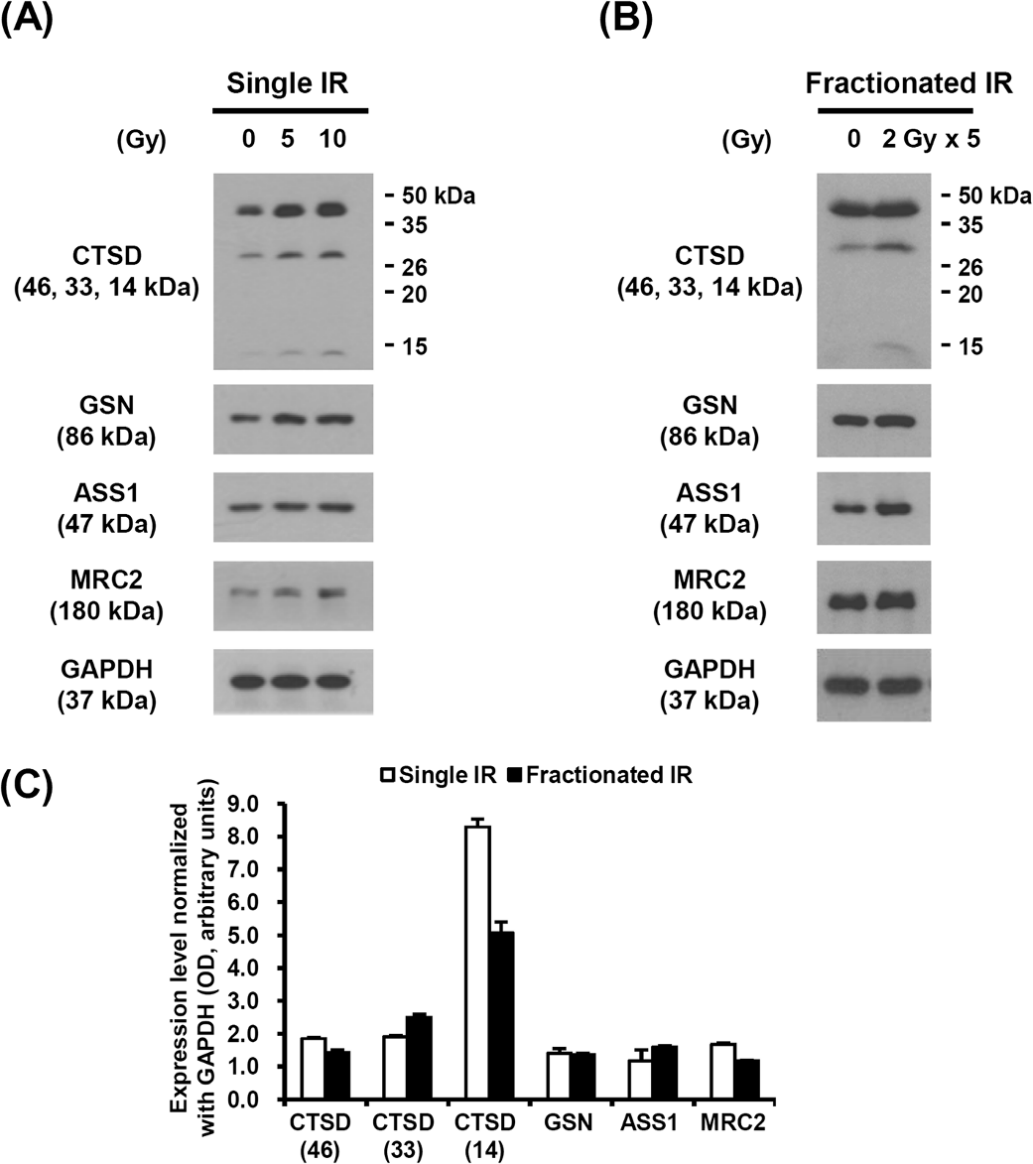
**Immunoblot analysis of candidate proteins.** MDA-MB-231 cells were exposed to the indicated single **(A)** or fractionated dose **(B)** of radiation. After 48 h, total protein was isolated and examined by western blot. **(C)** Band intensities corresponding to the indicated proteins were quantified by densitometry using ImageJ, normalized to the GAPDH loading control, and expressed as fold-change from each control. Values are mean ± SD for three independent experiments.

### Identification of potential proteins selected by quantitative proteomic analysis

Many previous reports have shown that treatment with ionizing radiation leads to an increase in FN1 expression and poor prognosis [23–25]. Thus, the expression of other candidate proteins after irradiation was investigated to confirm the results of the SILAC proteome approach. The expressions of CTSD and GSN were significantly induced by single-dose radiation in a dose-dependent manner, and the expressions of ASS1 and MRC2 were slightly increased (Figure 4A). Additionally, these four candidates were also significantly upregulated by the fractionated dose of ionizing radiation (Figure 4B). The expression level of candidate proteins induced by a single dose or multiple fractionated doses of irradiation was normalized to GAPDH expression. CTSD is synthesized as an inactive pre-proenzyme that is subsequently converted into an active, intermediate proenzyme (46 kDa) and matures after further cleavage into its more stable two-chain form (detected as 33 and 14 kDa). The bands corresponding to each stage of CTSD cleavage are analyzed in Figure 4C.

## Discussion

Radiation therapy is one of the most effective approaches for cancer treatment [1, 2]. However, radioresistance and recurrence are major obstacles for the long-term survival of patients undergoing radiation therapy [2, 8]. Thus, understanding the mechanisms of radiation-induced radioresistance and recurrence is important for the improvement of radiation therapy. In this study, SILAC-based quantitative proteomic analysis was performed to investigate the radiation-induced changes in protein expression in MDA-MB-231 cells with single or fractionated doses of radiation. Although several studies have analyzed protein profiles altered by radiation, this is the first report demonstrating and comparatively analyzing induction of altered molecules by single or fractionated irradiation. Previous studies have employed a single dose of 5–20 Gy radiation, but typical clinical fractionation schedules involve treatment with 1.5–2 Gy per day to a final 60–100 Gy exposure. Moreover, the efficacy of hypofractionated stereotactic body radiotherapy was recently evaluated and attracted attention as a promising curative radiotherapy option. Therefore, it is worthwhile to concurrently determine the proteomic profiles of cells treated with a single or fractionated dose of radiation.

Our results indicate the highest increase in FN1 expression, but the significance of this finding is modest due to different profiles after single or fractionated irradiation. Several studies have shown that ionizing radiation-induced FN1 is associated with radiation resistance, cell motility, and fibrosis [26–30]. Since FN1 expression may be greatly affected by fractionated radiation therapy, fractionated irradiation may cause more resistance than single dose irradiation. In various human cancers, especially breast cancer, high levels of CTSD expression were also reported [31]. Generally, CTSD plays a role in apoptosis, innate immune response, and inflammation but also stimulates growth, invasion, migration, angiogenesis [32, 33], and cellular senescence in cancer cells [34, 35]. Thus, the function of increased CTSD expression in cancer cells requires further investigation.

The interaction network of radiation-induced proteins was examined through STRING analysis. Interestingly, interactions between FN1, CTSD, GSN, and ASS1 were indicated, and these proteins were linked with ITGA4, ITGA5, and CD44. ITGA4 and ITGA5 are well-known fibronectin receptors that are altered in many cancer types [27, 36]. ITGA4 is overexpressed in chronic lymphocytic leukemia and associated with migration and retention in lymph node and bone marrow tissues. ITGA4 also promotes colony formation, drug resistance, and tumor-initiation in breast sarcomas [37, 38]. Similar to ITGA4, ITGA5 is involved in cell proliferation, survival, apoptosis, and angiogenesis [21, 27]. In addition, CD44 is responsible for cell adhesion, cell-cell interaction, lymphocyte activation, and tumor metastasis [39, 40]. GSN is a multifunctional actin-binding protein that controls the length of actin filaments through severing, capping, and nucleating activities. Changes in GSN level are observed in numerous human tumors and are closely related to higher migration capacity, development, and progression of cancer [41]. Therefore, we hypothesize that the FN1, CTSD, and GSN interaction is associated with cell adhesion and metastasis in tumor cells.

Several reports have suggested that MRC2 plays a role in extracellular matrix remodeling and metastasis [42, 43]. Through the STRING analysis, we found that MRC2 is linked with plasminogen activator system, TGF-β, and IL-10. In tumor tissues, plasminogen activator system is closely related to angiogenesis, fibrosis, and metastasis [44–46]. Moreover, TGF- β is also a well-known factor in immunosuppression, fibrosis, and the epithelial-mesenchymal transition (EMT) in tumor cells [47–49]. The function of anti-inflammatory cytokine IL-10 in tumor cells remains controversial as it has both tumor-suppressive and oncogenic roles [50, 51].

Several studies have demonstrated that migration and invasion are promoted by irradiation in breast cancer, pancreatic cancer, glioma, melanoma, rectal carcinoma, and colon carcinoma cells [52–58]. Our results suggest that radiation-induced increases of FN1, CTSD, GSN, and MRC2 may play radioresistant and negative roles in cancer therapy. Tumor cells killed by radiation treatment may release molecules unexpectedly but concomitantly produce favorable factors that protect undamaged tumor cells, thus increasing invasion or metastasis. However, the direct effect of those molecules on immune cells has not been investigated, so candidate proteins can possibly act as danger signals to stimulate the immune response for eradication of tumor cells after irradiation [16]. Further study is required to reveal the function of radiation-mediated protein expression in dendritic cells and provide good candidates for increasing radiosensitivity.

## Conclusion

Our results demonstrate that CTSD, GSN, and MRC2 are significantly increased in irradiated MDA-MB-231 cells and may be promising targets for predicting or enhancing the results of radiation therapy. Interest in combining radiotherapy with other immunotherapeutic agents, such as tumor vaccines, adoptive transfer of immune cells, and treatment with immune checkpoint blockers, is increasing. This study provides a front line approach for establishing the best condition for combined cancer therapeutics.

## Electronic supplementary material

Additional file 1: Figure S1: Evaluation of cytotoxicity by single or fractionated radiation. MDA-MB-231 cells were treated with single (10 Gy) or fractionated (2 Gy per day for 5 days) dose of radiation. Cell viability was determined by the MTT assay (A) or FACS analysis using propidium iodide (PI) fluorescent dye (B) 24, 48, or 72 h after the last irradiation. (TIFF 267 KB)

Additional file 2: Table S1: Up-regulated proteins were identified by a SILAC-based proteomic approach. **Table S2.** Down-regulated proteins were identified by a SILAC-based proteomic approach. (DOCX 37 KB)

## Abbreviations

ALDH2: Aldehyde dehydrogenase 2 family, mitochondrial
ASS1: Argininosuccinate synthase 1
CD44: Cluster of differentiation 44
CSPG4: Chondroitin sulfate proteoglycan 4
CTSD: Cathepsin D
DAMP: Damage-associated molecular patterns
EMT: Epithelial-mesenchymal transition
FBS: Fetal bovine serum
FN1: Fibronectin 1
GO: Gene ontology
GSN: Gelsolin
Gy: Gray
ICD: Immunogenic cell death
IL-10: Interleukin-10
ITGA4: Integrin alpha 4
ITGA5: Integrin alpha 5
LC-MS: Liquid chromatography–mass spectrometry
LRP1: Low-density lipoprotein receptor-related protein 1
MRC2: C-type mannose receptor 2
MTT: 3-[4,5-dimethylthiazol-2-yl]-2,5-diphenyltetrazolium bromide
NEDD4: Neural precursor cell expressed, developmentally down-regulated 4
PI: Propidium iodide
PRDX5: Peroxiredoxin 5
RT: Radiation therapy
SILAC: Stable isotope labeling by amino acids in cell culture
TBS-T: Tris-buffered saline containing 0.1% Tween-20
TGF-β: Transforming growth factor-beta.
