# Supplementary material for: Quantitative proteomic analysis of single or fractionated radiation-induced proteins in human breast cancer MDA-MB-231 cells
Source: Cell Biosci. 2015 Feb 3;5:2. doi: 10.1186/2045-3701-5-2 (PMC4459121; doi:10.1186/2045-3701-5-2)
Supplement: Supplementary file 2 — Additional file 2: Table S1: Up-regulated proteins were identified by a SILAC-based proteomic approach. Table S2. Down-regulated proteins were identified by a SILAC-based proteomic approach. (DOCX 37 KB) [file 13578_2014_212_MOESM2_ESM.docx]

Supplementary Table 1. Up-regulated proteins were identified by a SILAC-based proteomic approach

| **No** | **Accession**  **No** | **Gene Symbol** | **Identified Proteins** | **Fold** | ***P* value*** | **S 1** | **S 2** | **F 1** | **F 2** |
| --- | --- | --- | --- | --- | --- | --- | --- | --- | --- |
| 1 | IPI00022418 | FN1 | Isoform 1 of Fibronectin | 6.525 | 0.1451 | 1.7 | 1.9 | 17.7 | 4.8 |
| 2 | **IPI00011229** | **CTSD** | **Cathepsin D** | **3.200** | **0.0000** | **2.9** | **3.2** | **3.4** | **3.3** |
| 3 | **IPI00026314** | **GSN** | **Isoform 1 of Gelsolin** | **2.375** | **0.0000** | **3.0** | **2.6** | **2.0** | **1.9** |
| 4 | **IPI00020632** | **ASS1** | **Argininosuccinate synthase** | **2.175** | **0.0000** | **2.3** | **2.1** | **2.4** | **1.9** |
| 5 | IPI00024915 | PRDX5 | Isoform Mitochondrial of Peroxiredoxin-5, mitochondrial | 2.025 | 0.0007 | 2.9 | 1.8 | 1.5 | 1.9 |
| 6 | IPI00019157 | CSPG4 | Chondroitin sulfate proteoglycan 4 | 1.900 | 0.0606 | 1.4 | 1.0 | 2.0 | 3.2 |
| 7 | IPI00218474 | ENO3 | Isoform 1 of Beta-enolase | 1.900 | 0.3173 | 1.0 | 1.0 | 4.6 | 1.0 |
| 8 | **IPI00005707** | **MRC2** | **C-type mannose receptor 2** | **1.850** | **0.0145** | **1.3** | **1.2** | **2.5** | **2.4** |
| 9 | IPI00940829 | NEDD4 | Isoform 4 of E3 ubiquitin-protein ligase NEDD4 | 1.850 | 0.0074 | 1.3 | 1.3 | 2.4 | 2.4 |
| 10 | IPI00021048 | MYOF | Isoform 1 of Myoferlin | 1.800 | 0.0004 | 1.8 | 1.7 | 2.4 | 1.3 |
| 11 | IPI00021812 | AHNAK | Neuroblast differentiation-associated protein AHNAK | 1.800 | 0.0000 | 1.8 | 2.2 | 1.6 | 1.6 |
| 12 | IPI00020557 | LRP1 | Prolow-density lipoprotein receptor-related protein 1 | 1.800 | 0.0047 | 1.4 | 1.4 | 1.8 | 2.6 |
| 13 | IPI00021766 | RTN4 | Isoform 1 of Reticulon-4 | 1.725 | 0.0000 | 1.6 | 1.5 | 1.7 | 2.1 |
| 14 | IPI00006663 | ALDH2 | Aldehyde dehydrogenase, mitochondrial | 1.625 | 0.0019 | 2.1 | 1.8 | 1.4 | 1.2 |
| 15 | IPI00293464 | DDB1 | DNA damage-binding protein 1 | 1.475 | 0.0262 | 1.2 | 1.2 | 1.4 | 2.1 |
| 16 | IPI00007682 | ATP6V1A | V-type proton ATPase catalytic subunit A | 1.475 | 0.0009 | 1.1 | 1.4 | 1.7 | 1.7 |
| 17 | IPI00815770 | SNX3 | Isoform 1 of Sorting nexin-3 | 1.475 | 0.0841 | 1.2 | 1.2 | 2.3 | 1.2 |
| 18 | IPI00216694 | PLS3 | Plastin-3 | 1.450 | 0.0000 | 1.6 | 1.5 | 1.4 | 1.3 |
| 19 | IPI00098902 | OGDH | 2-oxoglutarate dehydrogenase, mitochondrial | 1.425 | 0.0000 | 1.5 | 1.5 | 1.4 | 1.3 |
| 20 | IPI00216457 | HIST2H2AA3 | Histone H2A type 2-A | 1.425 | 0.3447 | 0.6 | 0.7 | 2.3 | 2.1 |
| 21 | IPI00784156 | AP2B1 | Isoform 1 of AP-2 complex subunit beta | 1.425 | 0.0000 | 1.5 | 1.5 | 1.3 | 1.4 |
| 22 | IPI00010418 | MYO1C | Isoform 2 of Myosin-Ic | 1.425 | 0.0012 | 1.2 | 1.3 | 1.8 | 1.4 |
| 23 | IPI00018398 | PSMC3 | 26S protease regulatory subunit 6A | 1.400 | 0.3956 | 0.8 | 0.9 | 2.8 | 1.1 |
| 24 | IPI00000005 | NRAS | GTPase NRas | 1.400 | 0.0011 | 1.3 | 1.1 | 1.6 | 1.6 |
| 25 | IPI00328257 | AP1B1 | Isoform A of AP-1 complex subunit beta-1 | 1.400 | 0.0000 | 1.4 | 1.3 | 1.5 | 1.4 |
| 26 | IPI00026689 | CDK1 | Putative uncharacterized protein DKFZp686L20222 | 1.400 | 0.0000 | 1.4 | 1.2 | 1.5 | 1.5 |
| 27 | IPI00382844 | ACO2 | Aconitase (Fragment) | 1.375 | 0.0007 | 1.5 | 1.6 | 1.3 | 1.1 |
| 28 | IPI00246058 | PDCD6IP | Programmed cell death 6-interacting protein | 1.375 | 0.0015 | 1.2 | 1.2 | 1.7 | 1.4 |
| 29 | IPI00456969 | DYNC1H1 | Cytoplasmic dynein 1 heavy chain 1 | 1.350 | 0.0001 | 1.5 | 1.5 | 1.2 | 1.2 |
| 30 | IPI00013895 | S100A11 | Protein S100-A11 | 1.350 | 0.0000 | 1.4 | 1.5 | 1.3 | 1.2 |
| 31 | IPI00013860 | HIBADH | 3-hydroxyisobutyrate dehydrogenase, mitochondrial | 1.325 | 0.0135 | 1.5 | 1.6 | 1.1 | 1.1 |
| 32 | IPI00018350 | MCM5 | DNA replication licensing factor MCM5 | 1.325 | 0.0135 | 1.6 | 1.5 | 1.1 | 1.1 |
| 33 | IPI00001960 | CLIC4 | Chloride intracellular channel protein 4 | 1.325 | 0.0034 | 1.2 | 1.1 | 1.4 | 1.6 |
| 34 | IPI00289334 | FLNB | Isoform 1 of Filamin-B | 1.325 | 0.0000 | 1.4 | 1.4 | 1.2 | 1.3 |
| 35 | IPI00019502 | MYH9 | Isoform 1 of Myosin-9 | 1.325 | 0.0000 | 1.3 | 1.3 | 1.4 | 1.3 |
| 36 | IPI00011454 | GANAB | Isoform 2 of Neutral alpha-glucosidase AB | 1.325 | 0.0000 | 1.5 | 1.4 | 1.2 | 1.2 |
| 37 | IPI00215743 | RRBP1 | Isoform 3 of Ribosome-binding protein 1 | 1.325 | 0.0705 | 1.1 | 1.4 | 1.0 | 1.8 |
| 38 | IPI00006482 | ATP1A1 | Isoform Long of Sodium/potassium-transporting ATPase subunit alpha-1 | 1.325 | 0.0705 | 1.1 | 1.0 | 1.4 | 1.8 |
| 39 | IPI00026546 | PAFAH1B2 | Platelet-activating factor acetylhydrolase IB subunit beta | 1.325 | 0.0001 | 1.4 | 1.1 | 1.3 | 1.5 |
| 40 | IPI00009368 | SFXN1 | Sideroflexin-1 | 1.325 | 0.0000 | 1.4 | 1.3 | 1.3 | 1.3 |
| 41 | IPI00075248 | CALM3 | Calmodulin | 1.300 | 0.0002 | 1.3 | 1.5 | 1.1 | 1.3 |
| 42 | IPI00020984 | CANX | cDNA FLJ55574, highly similar to Calnexin | 1.300 | 0.0000 | 1.3 | 1.2 | 1.2 | 1.5 |
| 43 | IPI00180675 | TUBA1A | Tubulin alpha-1A chain | 1.300 | 0.0055 | 1.5 | 1.4 | 1.3 | 1.0 |
| 44 | IPI00003949 | UBE2N | Ubiquitin-conjugating enzyme E2 N | 1.300 | 0.0000 | 1.3 | 1.5 | 1.2 | 1.2 |
| 45 | IPI00023006 | ACTC1 | Actin, alpha cardiac muscle 1 | 1.300 | 0.2624 | 1.0 | 1.0 | 2.1 | 1.1 |
| 46 | IPI00013808 | ACTN4 | Alpha-actinin-4 | 1.300 | 0.0000 | 1.3 | 1.3 | 1.2 | 1.4 |

Proteins increased by an average of 1.3-fold or more are listed.

*Statistical comparisons were performed using the paired *z*-test, and experimentally verified proteins are represented in bold. S: single-dose irradiation, F; fractionated-dose irradiation.

Supplementary Table 2. Down-regulated proteins were identified by a SILAC-based proteomic approach

| no | Accession No | Gene  Symbol | Identified Proteins | Fold | *P* value | S 1 | S 2 | F 1 | F 2 |
| --- | --- | --- | --- | --- | --- | --- | --- | --- | --- |
| 1 | IPI00221091 | RPS15A | 40S ribosomal protein S15a | -1.600 | 0.0000 | 0.6 | 0.6 | 0.6 | 0.7 |
| 2 | IPI00221088 | RPS9 | 40S ribosomal protein S9 | -1.600 | 0.0000 | 0.6 | 0.6 | 0.7 | 0.6 |
| 3 | IPI00306332 | RPL24 | 60S ribosomal protein L24 | -1.538 | 0.0000 | 0.6 | 0.7 | 0.6 | 0.7 |
| 4 | IPI00182533 | RPL28 | 60S ribosomal protein L28 | -1.538 | 0.0000 | 0.7 | 0.6 | 0.7 | 0.6 |
| 5 | IPI00026302 | RPL31 | 60S ribosomal protein L31 | -1.538 | 0.0000 | 0.7 | 0.6 | 0.7 | 0.6 |
| 6 | IPI00029750 | RPS24 | Isoform 1 of 40S ribosomal protein S24 | -1.538 | 0.0000 | 0.6 | 0.6 | 0.7 | 0.7 |
| 7 | IPI00026202 | RPL18A | 60S ribosomal protein L18a | -1.538 | 0.0000 | 0.7 | 0.7 | 0.6 | 0.6 |
| 8 | IPI00025091 | RPS11 | 40S ribosomal protein S11 | -1.481 | 0.0000 | 0.6 | 0.7 | 0.7 | 0.7 |
| 9 | IPI00221089 | RPS13 | 40S ribosomal protein S13 | -1.481 | 0.0000 | 0.7 | 0.6 | 0.7 | 0.7 |
| 10 | IPI00217030 | RPS4X | 40S ribosomal protein S4, X isoform | -1.481 | 0.0000 | 0.7 | 0.6 | 0.7 | 0.7 |
| 11 | IPI00027270 | RPL26 | 60S ribosomal protein L26 | -1.481 | 0.0000 | 0.7 | 0.7 | 0.7 | 0.6 |
| 12 | IPI00219155 | RPL27 | 60S ribosomal protein L27 | -1.481 | 0.0000 | 0.6 | 0.7 | 0.7 | 0.7 |
| 13 | IPI00029731 | RPL35A | 60S ribosomal protein L35a | -1.481 | 0.0000 | 0.6 | 0.7 | 0.7 | 0.7 |
| 14 | IPI00008438 | RPS10 | 40S ribosomal protein S10 | -1.481 | 0.0000 | 0.7 | 0.7 | 0.6 | 0.7 |
| 15 | IPI00444262 | NCL | cDNA FLJ45706 fis, clone FEBRA2028457, highly similar to Nucleolin | -1.481 | 0.0000 | 0.6 | 0.6 | 0.7 | 0.8 |
| 16 | IPI00001734 | PSAT1 | Phosphoserine aminotransferase | -1.481 | 0.0000 | 0.6 | 0.6 | 0.8 | 0.7 |
| 17 | IPI00013917 | RPS12 | 40S ribosomal protein S12 | -1.429 | 0.0000 | 0.7 | 0.7 | 0.7 | 0.7 |
| 18 | IPI00221092 | RPS16 | 40S ribosomal protein S16 | -1.429 | 0.0000 | 0.7 | 0.7 | 0.7 | 0.7 |
| 19 | IPI00221093 | RPS17 | 40S ribosomal protein S17 | -1.429 | 0.0000 | 0.7 | 0.6 | 0.7 | 0.8 |
| 20 | IPI00013296 | RPS18 | 40S ribosomal protein S18 | -1.429 | 0.0000 | 0.7 | 0.7 | 0.7 | 0.7 |
| 21 | IPI00215780 | RPS19 | 40S ribosomal protein S19 | -1.429 | 0.0000 | 0.7 | 0.7 | 0.7 | 0.7 |
| 22 | IPI00012750 | RPS25 | 40S ribosomal protein S25 | -1.429 | 0.0000 | 0.7 | 0.7 | 0.7 | 0.7 |
| 23 | IPI00746004 | RPS27L | 40S ribosomal protein S27-like | -1.429 | 0.0000 | 0.6 | 0.7 | 0.7 | 0.8 |
| 24 | IPI00011253 | RPS3 | 40S ribosomal protein S3 | -1.429 | 0.0000 | 0.7 | 0.7 | 0.7 | 0.7 |
| 25 | IPI00008433 | RPS5 | 40S ribosomal protein S5 | -1.429 | 0.0000 | 0.7 | 0.7 | 0.7 | 0.7 |
| 26 | IPI00216587 | RPS8 | 40S ribosomal protein S8 | -1.429 | 0.0000 | 0.7 | 0.7 | 0.7 | 0.7 |
| 27 | IPI00008530 | RPLP0 | 60S acidic ribosomal protein P0 | -1.429 | 0.0000 | 0.7 | 0.7 | 0.7 | 0.7 |
| 28 | IPI00412579 | RPL10A | 60S ribosomal protein L10a | -1.429 | 0.0000 | 0.7 | 0.7 | 0.7 | 0.7 |
| 29 | IPI00219156 | RPL30 | 60S ribosomal protein L30 | -1.429 | 0.0000 | 0.7 | 0.7 | 0.7 | 0.7 |
| 30 | IPI00395998 | RPL32 | 60S ribosomal protein L32 | -1.429 | 0.0000 | 0.7 | 0.7 | 0.7 | 0.7 |
| 31 | IPI00000494 | RPL5 | 60S ribosomal protein L5 | -1.429 | 0.0000 | 0.7 | 0.7 | 0.7 | 0.7 |
| 32 | IPI00012772 | RPL8 | 60S ribosomal protein L8 | -1.429 | 0.0000 | 0.7 | 0.6 | 0.8 | 0.7 |
| 33 | IPI00031691 | RPL9 | 60S ribosomal protein L9 | -1.429 | 0.0000 | 0.7 | 0.7 | 0.7 | 0.7 |
| 34 | IPI00218606 | RPS23 | 40S ribosomal protein S23 | -1.429 | 0.0000 | 0.7 | 0.7 | 0.8 | 0.6 |
| 35 | IPI00304612 | RPL13A | 60S ribosomal protein L13a | -1.429 | 0.0000 | 0.7 | 0.7 | 0.8 | 0.6 |
| 36 | IPI00413324 | RPL17 | 60S ribosomal protein L17 | -1.379 | 0.0000 | 0.7 | 0.7 | 0.7 | 0.8 |
| 37 | IPI00216237 | RPL36 | 60S ribosomal protein L36 | -1.379 | 0.0000 | 0.7 | 0.7 | 0.7 | 0.8 |
| 38 | IPI00013485 | RPS2 | 40S ribosomal protein S2 | -1.379 | 0.0000 | 0.7 | 0.8 | 0.7 | 0.7 |
| 39 | IPI00012493 | RPS20 | 40S ribosomal protein S20 | -1.379 | 0.0000 | 0.7 | 0.7 | 0.8 | 0.7 |
| 40 | IPI00470528 | RPL15 | 60S ribosomal protein L15 | -1.379 | 0.0000 | 0.7 | 0.7 | 0.8 | 0.7 |
| 41 | IPI00010153 | RPL23 | 60S ribosomal protein L23 | -1.379 | 0.0000 | 0.7 | 0.7 | 0.8 | 0.7 |
| 42 | IPI00215790 | RPL38 | 60S ribosomal protein L38 | -1.379 | 0.0000 | 0.7 | 0.7 | 0.8 | 0.7 |
| 43 | IPI00013452 | EPRS | Bifunctional aminoacyl-tRNA synthetase | -1.379 | 0.0000 | 0.8 | 0.7 | 0.6 | 0.8 |
| 44 | IPI00848226 | GNB2L1 | Guanine nucleotide-binding protein subunit beta-2-like 1 | -1.379 | 0.0000 | 0.7 | 0.7 | 0.8 | 0.7 |
| 45 | IPI00024933 | RPL12 | Isoform 1 of 60S ribosomal protein L12 | -1.379 | 0.0000 | 0.7 | 0.7 | 0.8 | 0.7 |
| 46 | IPI00413108 | RPSA | 33 kDa protein | -1.333 | 0.0000 | 0.7 | 0.7 | 0.8 | 0.8 |
| 47 | IPI00026271 | RPS14 | 40S ribosomal protein S14 | -1.333 | 0.0000 | 0.7 | 0.7 | 0.8 | 0.8 |
| 48 | IPI00479058 | RPS15 | 40S ribosomal protein S15 | -1.333 | 0.0000 | 0.7 | 0.7 | 0.8 | 0.8 |
| 49 | IPI00013415 | RPS7 | 40S ribosomal protein S7 | -1.333 | 0.0000 | 0.7 | 0.8 | 0.7 | 0.8 |
| 50 | IPI00021266 | RPL23A | 60S ribosomal protein L23a | -1.333 | 0.0000 | 0.7 | 0.7 | 0.8 | 0.8 |
| 51 | IPI00299573 | RPL7A | 60S ribosomal protein L7a | -1.333 | 0.0000 | 0.7 | 0.8 | 0.8 | 0.7 |
| 52 | IPI00479186 | PKM | Isoform M2 of Pyruvate kinase isozymes M1/M2 | -1.333 | 0.0000 | 0.8 | 0.8 | 0.7 | 0.7 |
| 53 | IPI00002520 | SHMT2 | Serine hydroxymethyltransferase, mitochondrial | -1.333 | 0.0039 | 0.9 | 0.9 | 0.6 | 0.6 |

Proteins decreased by an average 1.3-fold or more are listed.

*Statistical comparisons were performed using the paired *z-*test, and experimentally verified proteins are represented in bold. S: single-dose irradiation, F; fractionated-dose irradiation.
